# Network-specific enhancement of global blood-oxygen-level-dependent signals and CSF coupling after language therapy in post-stroke aphasia

**DOI:** 10.1093/braincomms/fcag106

**Published:** 2026-03-24

**Authors:** Chao Zhang, Tao Feng, Jingyun Sha, Xiao Wang, Lulu Cai, Chong Meng, Chunfeng Hu, Jie Xiang, Kai Xu

**Affiliations:** Department of Radiology, Affiliated Hospital of Xuzhou Medical University, Xuzhou, Jiangsu Province 221006, P.R. China; Department of Rehabilitation, Affiliated Hospital of Xuzhou Medical University, Xuzhou, Jiangsu Province 221006, P.R. China; Department of Radiology, Affiliated Hospital of Xuzhou Medical University, Xuzhou, Jiangsu Province 221006, P.R. China; Department of Radiology, Affiliated Hospital of Xuzhou Medical University, Xuzhou, Jiangsu Province 221006, P.R. China; Department of Radiology, Affiliated Hospital of Xuzhou Medical University, Xuzhou, Jiangsu Province 221006, P.R. China; Department of Radiology, Affiliated Hospital of Xuzhou Medical University, Xuzhou, Jiangsu Province 221006, P.R. China; Department of Radiology, Affiliated Hospital of Xuzhou Medical University, Xuzhou, Jiangsu Province 221006, P.R. China; Department of Rehabilitation, Affiliated Hospital of Xuzhou Medical University, Xuzhou, Jiangsu Province 221006, P.R. China; Department of Radiology, Affiliated Hospital of Xuzhou Medical University, Xuzhou, Jiangsu Province 221006, P.R. China

**Keywords:** post-stroke aphasia, cerebrospinal fluid, resting-state fMRI, BOLD, network

## Abstract

Emerging evidence indicates that stroke may impair glymphatic function by disrupting cerebrospinal fluid clearance. The coupling between global blood-oxygen-level-dependent signals and cerebrospinal fluid flow has been proposed as a non-invasive biomarker of glymphatic activity. However, it remains unclear whether this coupling can be modulated through rehabilitation. This study investigated whether language therapy in patients with post-stroke aphasia enhances global blood-oxygen-level-dependent– cerebrospinal fluid coupling, thereby reflecting potential restoration of fluid–brain interaction. This longitudinal observational study was conducted at a single centre and included 20 patients with post-stroke aphasia and 35 age- and sex-balanced healthy controls. All participants underwent MRI scanning, including resting-state blood-oxygen-level-dependent and structural imaging. Among the post-stroke aphasia group, 14 patients completed both pre- and post-treatment assessments after undergoing a standardized 4-week speech-language therapy programme. The Western Aphasia Battery was used to quantify language deficits and monitor treatment-related changes. Global blood-oxygen-level-dependent–cerebrospinal fluid coupling was quantified using cross-correlation analysis across the whole brain and four predefined language-related resting-state networks. At baseline, patients exhibited significantly reduced global blood-oxygen-level-dependent–cerebrospinal fluid coupling compared to healthy controls (*P* < 0.05). Following therapy, coupling significantly increased within the language, salience and dorsal attention networks, whereas coupling within the default mode network significantly decreased (all *P* < 0.05). Notably, increased global blood-oxygen-level-dependent–cerebrospinal fluid coupling in specific networks was significantly correlated with improvements in targeted language functions, such as object naming, responsive naming and auditory word recognition (*P* < 0.05). These findings suggest that language rehabilitation enhances neurophysiological coupling between brain activity and cerebrospinal fluid flow, potentially reflecting restoration of fluid–brain interaction in post-stroke aphasia.

## Teaser

Zhang *et al*. report that language therapy in patients with post-stroke aphasia enhances coupling between global blood oxygen level–dependent brain activity and cerebrospinal fluid dynamics, particularly within language-related brain systems. These findings suggest that coordinated interactions between brain activity and cerebrospinal fluid circulation may contribute to mechanisms underlying language recovery after stroke.

## Graphical Abstract

**Graphical Abstract:**
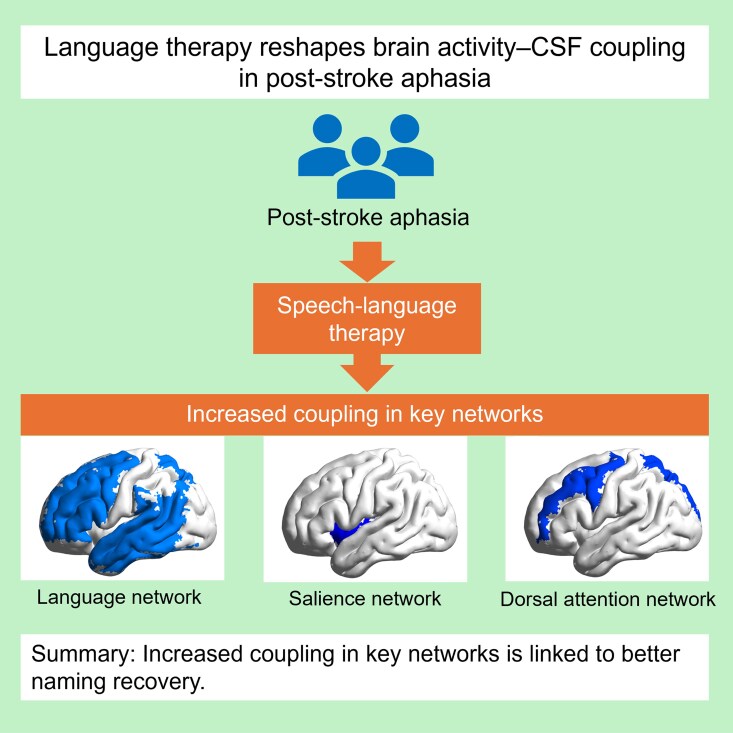

## Introduction

Stroke remains a leading cause of long-term disability and mortality globally, imposing a substantial burden on healthcare systems and society.^1^ Among its sequelae, post-stroke aphasia (PSA) is one of the most debilitating, affecting approximately 30% of survivors.^2^ Although standard rehabilitation can partially restore language ability, many patients experience persistent deficits. Growing evidence suggests that PSA arises from not only focal cortical damage but also from large-scale network dysfunction and altered neurovascular regulation.^3^

Recent neuroimaging studies have shifted focus from lesion-centred to network-level perspectives.^4-7^ Resting-state fMRI has been instrumental in revealing altered activity both within and beyond traditional language areas, and network-based metrics such as efficiency and modularity have shown promise in predicting treatment response.^6^ Complementary techniques, such as arterial spin labelling and amplitude-based measures, further corroborate the presence of widespread neural reorganization during recovery.^5,8,9^ In particular, the integrity and interaction of networks such as the default mode network (DMN), executive control network and salience network (SN), shape multidomain cognitive outcomes after stroke.^7^ Similarly, recovery from PSA reflects reorganization across fronto-temporo-parietal pathways, right-hemisphere homologues, and domain-general systems including the SN and dorsal attention networks (DAN) that support attentional control and resource allocation.^10-12^These convergent observations highlight a network-based framework for PSA, emphasizing that rehabilitation involves coordinated reconfiguration of multiple systems rather than isolated regional repair. Yet, a unified physiological model linking these network-level changes to behavioural recovery remains unclear.

In light of these interpretive challenges, investigating physiological mechanisms that may underlie or modulate network-level alteration in PSA represents a promising area of inquiry. The glymphatic system, a brain-wide clearance pathway facilitating cerebrospinal fluid (CSF)–interstitial fluid (ISF) exchange, plays a key role in maintaining neural homeostasis^13-19^ Experimental studies have demonstrated that stroke can disrupt glymphatic function, resulting in impaired CSF influx, reduced interstitial solute clearance, and loss of aquaporin-4 polarization on astrocytic endfeet.^20,21^  *In vivo* evidence from diffusion tensor imaging along perivascular spaces (DTI-ALPS) and contrast-enhanced MRI supports the presence of glymphatic impairment after stroke, with partial recovery observed over time.^15^ Such dysfunction has also been implicated in secondary injury mechanisms, including oedema, neuroinflammation and cognitive decline.^13,20^ However, metrics such as the DTI-ALPS index may be confounded by post-stroke structural changes and deep white matter artefacts,^22,23^ underscoring the need for alternative physiological markers.

Global blood-oxygen-level-dependent (gBOLD) signal–CSF coupling, an fMRI-based approach proposed by Fultz *et al*.,^16,24-26^ quantifies the temporal interaction between global neural activity and CSF inflow and may indirectly reflect neurofluid and glymphatic dynamics. Recent studies have extended this measure to small-vessel and Moyamoya disease, linking coupling alterations to vascular and CSF regulatory dysfunctions.^27,28^ However, its relevance to PSA and therapy-related recovery has not been examined.

In this study, we applied gBOLD–CSF coupling to investigate its changes following language rehabilitation in PSA. We hypothesized that therapy would increase coupling, particularly within language-related networks. By linking network-specific neurofluid coupling dynamics with behavioural gains, this work aims to provide a novel physiological framework for understanding recovery mechanisms in PSA.

## Materials and methods

### Participants

The retrospective study analysed data from 23 patients with PSA, diagnosed 1 month after their initial stroke. Among them, 14 patients received 1 month of speech-language therapy (SLT), and completed clinical as well as MRI follow-up evaluations. Inclusion criteria for these patients were as follows: (i) lesion localized in the left cerebral hemisphere, as verified by MRI; (ii) first-ever stroke with no prior history of symptomatic stroke or other major neurological disorders; (iii) right-handedness, to minimize variability in language lateralization; (iv) stable general medical condition; (v) no history of cognitive impairment prior to the stroke; (vi) sufficient alertness and cooperation to complete MRI scans, clinical evaluations and rehabilitation sessions. Exclusion criteria included MRI findings of other brain abnormalities like tumours or haemangiomas, a history of head trauma, psychiatric disorders or MRI contraindications. Patients presenting with severe medical conditions that could impede rehabilitation—such as coma, cardiorespiratory failure or significant physical impairments that hinder treatment cooperation were excluded from the study. Concurrently, 35 healthy controls (HCs) with no history of neurological or psychiatric disorders were included, ensuring a comparable age and sex distribution to the PSA group. Exclusion criteria for the controls included the presence of MRI-detected intracranial lesions and a family history of hereditary or neurological diseases.

The study was conducted in accordance with the 1964 Declaration of Helsinki. Ethical approval was obtained from the Ethics Committee of Affiliated Hospital of Xuzhou Medical University (No. XYFY2017-KL037-02). The requirement for informed consent was waived due to the retrospective nature of the study.

### Clinical assessment and speech-language therapy

All PSA patients underwent clinical evaluation and MRI scans before and after completing a 4-week SLT programme. To assess aphasia severity and expressive language function, the Chinese version of the Western Aphasia Battery (WAB) was used, ensuring cultural and linguistic relevance. All assessments were conducted by certified speech and language therapists following standardized protocols.

The SLT programme was tailored to individual patient needs based on their WAB ratings. The rehabilitation regimen included structured exercises targeting core language skills, such as listening, speaking, reading and writing, with progressive difficulty levels spanning words, phrases and sentences. Patients participated in two 30-min therapy sessions per day, 6 days per week, for 4 weeks. The intervention aimed to enhance functional language recovery by addressing both phonological and semantic processing deficits.^29^

### MRI data acquisition

All participants underwent identical MRI scanning protocols using a 3.0T GE Signa HD system (GE Medical Systems, Waukesha, WI, USA) equipped with an eight-channel phased-array head coil. Imaging for PSA patients was conducted before and after the 4-week SLT, aligning with their WAB assessments. HCs underwent a single MRI session. To exclude additional brain abnormalities, whole-brain axial FLAIR imaging was performed. High-resolution T1WI was acquired using a 3D T1 BRAVO sequence, providing isotropic voxels of 1 mm^3^ for detailed structural assessment. BOLD data were collected using an echo-planar imaging sequence covering the whole brain. The acquisition parameters comprised 36 slices with a matrix size of 64 × 64, a slice thickness/gap of 3 mm/1 mm, a repetition time of 2000ms, an echo time of 30 ms, a flip angle of 90° and a total of 185 time points. All MRI scans were performed during daytime hours, with most sessions conducted between 8:00 and 12:00 AM, with the remainder completed before 5:00 PM, to minimize circadian variability in CSF dynamics.

### Lesion mapping

**Figure 1:**
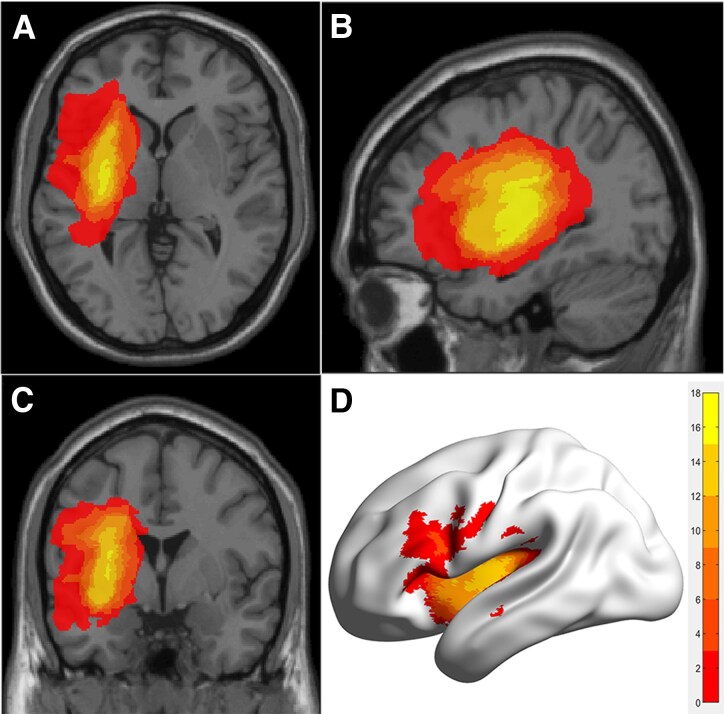
**Lesion overlap maps of all patients with post-stroke aphasia (*n* = 20).** (A–C) Axial (**A**), sagittal (**B**), and coronal (**C**) slices showing lesion overlap across patients, overlaid on the MNI152 template. The colour scale reflects the number of patients with overlapping lesions, with yellow indicating the highest degree of overlap. (**D**) 3D surface rendering of the lesion distribution on the inflated cortical surface of the left hemisphere. The colour bar denotes the number of patients overlapping at each voxel (range: 2–18).

Lesions were manually delineated on high-resolution 3DT1WI using ITK-SNAP^5^(Fig. 1) by two board-certified radiologists (J.S. and X.W.). For each patient, ITK-SNAP was used to manually delineate the lesion, after which the software automatically generated both the lesion volume and a binary mask for subsequent spatial normalization, as described in our previous study.^5^ All delineations were reviewed by a senior neuroradiologist, and a random subset was cross-checked between raters to confirm high consistency. Lesion volumes were calculated to assess potential heterogeneity across patients, and no significant correlations were found between lesion size and gBOLD–CSF coupling across networks (all *P* > 0.05; see Supplementary Table 1).

### Functional data preprocessing

Resting-state fMRI (rs-fMRI) data were preprocessed and analysed using the DPARSF 5.4 toolbox (Data Processing Assistant for Resting-State fMRI) within the MATLAB (MathWorks, Natick, MA, USA) environment.^30^ To ensure methodological rigour and comparability, distinct preprocessing pipelines were implemented for different signal analyses.

For the gBOLD signal, preprocessing included slice timing correction, followed by nuisance regression to remove confounding signals from white matter, CSF, and head motion parameters, using the Friston-24 model. Additionally, detrending and bandpass filtering (0.01–0.1 Hz) were applied to retain low-frequency fluctuations relevant to resting-state dynamics.

For the analysis of CSF fluctuations, a more selective approach was employed to preserve signal characteristics related to CSF dynamics. In accordance with previously established methodologies,^16,31^ only slice timing correction, detrending and bandpass filtering (0.01–0.1 Hz) were applied, without regressing out CSF signals. This approach ensured that CSF-related signal fluctuations were preserved, thereby allowing for a more precise evaluation of CSF flow patterns.

### Coupling between gBOLD signal and CSF flow

**Figure 2:**
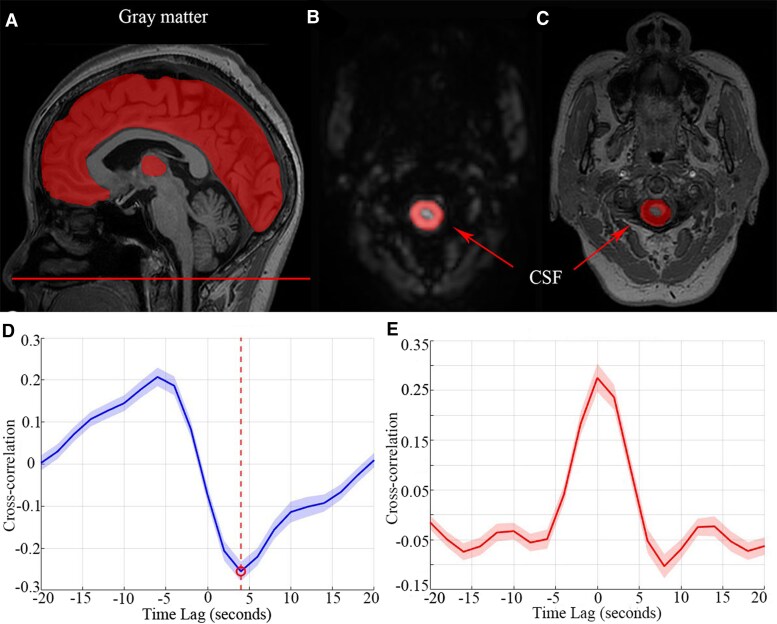
**gBOLD–CSF signal extraction and cross-correlation analysis.** (**A**) The gBOLD signal was extracted from grey matter regions defined by the Harvard-Oxford cortical and subcortical atlases (highlighted in red). (**B**, **C**) The CSF signal was extracted from the bottom fMRI slice near the cerebellum (red-highlighted region). (**D**) Cross-correlation analysis between the gBOLD and CSF signals revealed a distinct negative peak at approximately +4 s (*r* = −0.26, *P* < 0.001, Pearson correlation, *n* = 55). (**E**) Cross-correlation between the CSF signal and the negative first-order derivative of the gBOLD signal showed a positive peak at zero lag (*r* = 0.28, *P* < 0.001, Pearson correlation, *n* = 55).

Grey matter gBOLD signals were extracted from regions defined by the Harvard-Oxford cortical and subcortical atlases (Fig. 2A). Atlas masks defined in MNI space were first warped to each participant’s native 3D T1-weighted image using inverse deformation fields from SPM12, and then coregistered to the mean fMRI image to obtain subject-specific masks in native fMRI space. The fMRI signal was z-normalized and averaged across all grey matter voxels to assess global brain activity. The CSF signal was obtained from the lowest fMRI slice near the cerebellum, where inflow effects are most pronounced (Fig. 2B and C). This region was manually delineated based on fMRI hyperintensity and verified using T1-weighted images. CSF ROIs were delineated on the bottom fMRI slice using the same standardized protocol as lesion delineation. All masks were reviewed by a senior neuroradiologist, and a random subset was cross-checked between raters to ensure consistent definition of the CSF region. The CSF signal was normalized to its temporal mean. To quantify the coupling between gBOLD and CSF signals, we calculated the cross-correlation function. Coupling strength was determined at a + 4-s time lag. Additionally, for comparative purposes, we computed the cross-correlation between the negative first-order derivative of the gBOLD signal and the CSF signal.^16,31^ In addition to whole-brain analysis, gBOLD–CSF coupling was further evaluated within four resting-state networks defined by anatomical labels from the AAL3 atlas: the DMN, SN, DAN and language network (LN). These networks were selected based on their previously reported involvement in language processing and recovery, as described in task-free fMRI literature.^6^ Individualized binary masks were generated for each subject to extract network-specific mean coupling values at both pre- and post-treatment timepoints.

### Statistics

All statistical analyses were conducted using MATLAB R2023b. Continuous variables were tested for normality using the Shapiro–Wilk test. Between-group comparisons between PSA patients and HCs were performed using linear regression models, adjusting for age and sex as covariates. Longitudinal comparisons of gBOLD–CSF coupling before and after treatment, as well as comparisons with HCs, were assessed using linear mixed-effects models with condition as a fixed effect and subject as a random intercept to account for paired measurements in patients. Post hoc pairwise comparisons were performed with Holm correction for multiple comparisons. To examine the association between changes in gBOLD–CSF coupling and improvements in language performance, linear regression models were applied, adjusting for age and baseline language scores and lesion volume as covariates. All *P* values were two-tailed, and statistical significance was defined as *P* < 0.05. For analyses involving multiple networks or language-related measures, *P* values were corrected for multiple comparisons (FDR, q < 0.05). The experimental unit for all statistical tests was the individual participant. No blinding or randomization was applied due to the observational nature of this longitudinal study. However, all image preprocessing and statistical pipelines were applied uniformly to minimize procedural bias. A priori sample size estimation (α = 0.05, power = 80%) based on previous literature suggested that the current sample size was sufficient to detect medium-to-large effects. No pseudo-replication was introduced, as all analyses were conducted at the participant level. As this was a retrospective study, only patients with complete clinical and imaging datasets were included. Therefore, no missing data handling procedures were required.

### Statistical power analysis

Statistical power analysis was performed in MATLAB R2023b, utilizing Cohen’s *d* effect sizes. Specifically, a power analysis for paired *t*-tests was conducted with an α level of 0.05 (two-tailed) and a target statistical power of 80%. Effect sizes were derived from the differences observed between pre- and post-treatment conditions, and the required sample sizes were estimated to ensure adequate power. Effect sizes varied across linguistic assessments, with the largest (*d* = 1.35–1.38) necessitating the smallest sample sizes (*n* = 6), while smaller effects (*d* = 0.72) required larger samples (*n* = 11). A total of 14 patients completed the pre- and post-treatment assessments, thereby meeting the power requirements. For gBOLD-CSF coupling, Cohen's *d* was calculated to be 0.55 (PSA versus HC) and 0.67 (PSA pre- versus post-treatment), indicating moderate to large effects. The power analysis confirmed that the sample size was sufficient to achieve 80% power at α = 0.05, thereby ensuring the robustness of the statistical findings.

### Outcome and variable definition

The primary outcome was the change in language performance, assessed by speech fluency, information content, yes/no questions, auditory word recognition, sequential order, repetition, object naming, responsive naming, sentence completion and word fluency. We examined the associations between these changes and gBOLD–CSF coupling strength at the whole-brain and network levels. Age and baseline language performance were considered as potential confounding factors.

## Results

### Demographics and clinical characteristics

Initially, 23 PSA patients were included in this study. However, three patients were excluded due to excessive head motion or the presence of extensive intracranial lesions, resulting in a final sample size of 20 PSA patients and 35 HCs for analysis. The PSA cohort consisted of 11 men and 9 women, with an age range of 33 to 67 years (mean age: 46.50 ± 9.45 years), and the HC group included 19 men and 16 women, with an age range of 30–59 years (mean age: 48.00 ± 9.14 years). Statistical analysis revealed no significant differences in age (*P* = 0.57) or sex distribution (*P* > 0.99) between the two groups. A comprehensive overview of the demographic and clinical characteristics of all participants is presented in Table 1. Paired *t*-tests revealed significant improvements across all major WAB domains, including Spontaneous Speech, Comprehension, Repetition, and Naming (all *P* < 0.05), as well as in the overall Aphasia Quotient (AQ) (Table 2).

**Table 1:** Demographic characteristics of PSA patients and HCs

Characteristic	HCs (*N* = 35)	PSA (*N* = 20)	*P*-value^a^
Age, mean (SD)	48.00 (9.14)	46.50 (9.45)	0.57
Gender			>0.99
Male	19 (54.3%)	11 (55.0%)	
Female	16 (45.7%)	9 (45.0%)	

Values are presented as mean (SD) or number (%).

^a^ *P*-values are derived from two-sample *t*-tests or Pearson's chi-squared tests as appropriate.

**Table 2:** Comparison of WAB subtest scores before and after treatment

Subtest	Pre-treatment	Post-treatment	*T*	*P*-value^a^
**AQ**	46.13 ± 13.16	70.25 ± 10.30	9.606	<0.001
**Spontaneous**	7.13 ± 1.64	12.15 ± 2.01	5.212	<0.001
Speech fluency	3.14 ± 1.75	5.57 ± 2.31	3.426	0.005
Information content	4.00 ± 1.52	6.57 ± 1.16	4.746	<0.001
**Comprehension**	5.69 ± 1.39	7.63 ± 1.13	6.423	<0.001
Yes/no questions	45.29 ± 6.83	48.57 ± 8.83	1.674	0.118
Auditory word recognition	44.45 ± 8.49	53.58 ± 3.40	4.652	<0.001
Sequential order	25.57 ± 20.47	48.93 ± 17.89	5.120	<0.001
**Repetition**	7.17 ± 1.53	8.80 ± 1.04	4.603	<0.001
Repetition	71.72 ± 15.32	88.03 ± 10.44	4.603	<0.001
**Naming**	3.01 ± 1.47	6.53 ± 1.99	5.190	<0.001
Object naming	17.64 ± 12.00	48.29 ± 13.30	7.737	<0.001
Word fluency	5.71 ± 2.61	8.43 ± 2.21	3.153	0.008
Sentence completion	2.79 ± 2.36	6.86 ± 2.28	4.251	0.001
Responsive naming	1.29 ± 1.64	5.71 ± 3.17	4.460	0.001

Values are presented as mean (SD).

All WAB subscores represent standardized (converted) values according to the WAB manual. Each major domain (spontaneous, comprehension, repetition, naming) was scaled to a 0–10 range.

^a^ *P*-values are derived from paired *t*-tests.

### GBOLD signal is coupled to CSF signal changes

To examine the coupling between gBOLD and CSF signal fluctuations, we performed a cross-correlation analysis on both HC and PSA cohorts, following the methodology outlined by Fultz *et al*.^16^ Our analysis revealed a distinct negative peak at an approximate lag of +4 s (*r* = −0.26, *P* < 0.001) (Fig. 2D). Additionally, the cross-correlation between the CSF signal and the negative first-order derivative of the gBOLD signal exhibited a significant positive peak at zero time lag (*r* = 0.28, *P* < 0.001) (Fig. 2E), consistent with previous research findings.

### Comparison of gBOLD-CSF coupling Among groups

**Figure 3:**
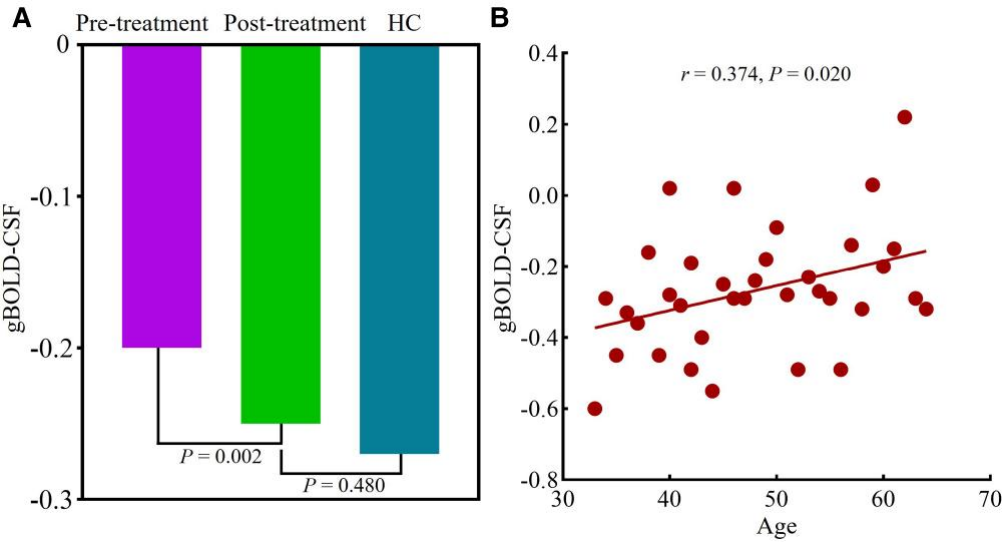
**Group differences and age association of gBOLD–CSF coupling in PSA patients and HCs.** (**A**) Data are shown for PSA patients at baseline (pre-treatment, *n* = 14), PSA patients after treatment (post-treatment, *n* = 14), and HCs (*n* = 35). Group differences were assessed using a linear mixed-effects model with condition (pre-treatment, post-treatment, and HC) as a fixed effect and subject as a random intercept to account for paired measurements in patients. Post hoc pairwise comparisons were performed with Holm correction for multiple comparisons. gBOLD–CSF coupling was significantly increased after treatment compared with baseline (estimated difference = 0.054, 95% CI 0.021–0.087, *P* = 0.002), and post-treatment coupling values were not significantly different from those of HCs (estimated difference = 0.018, 95% CI −0.032 to 0.067, *P* = 0.48). (**B**) Partial correlation analysis controlling for sex and lesion volume revealed a positive association between gBOLD–CSF coupling and age across all participants (*r* = 0.374, *P* = 0.020; *n* = 55). Each data point represents one participant.

We first assessed group-level differences in gBOLD–CSF coupling. In the full baseline cohort, PSA patients showed significantly reduced gBOLD–CSF coupling compared with HCs after adjustment for age and sex (*β* = 0.073*, P* = 0.024). In longitudinal analyses using a linear mixed-effects model, gBOLD–CSF coupling significantly increased after treatment compared with baseline (estimated difference = 0.054, 95% CI 0.021–0.087, *P* = 0.002). Post-treatment coupling values were not significantly different from those of HCs (estimated difference = 0.018, 95% CI −0.032 to 0.067, *P* = 0.480; Fig. 3A). Age was included as a covariate in comparisons between PSA and HC groups, given a significant positive correlation between age and gBOLD–CSF coupling (*r* = 0.374, *P* = 0.020; Fig. 3B).

### Network-specific changes and clinical correlates of gBOLD–CSF coupling

**Figure 4.**
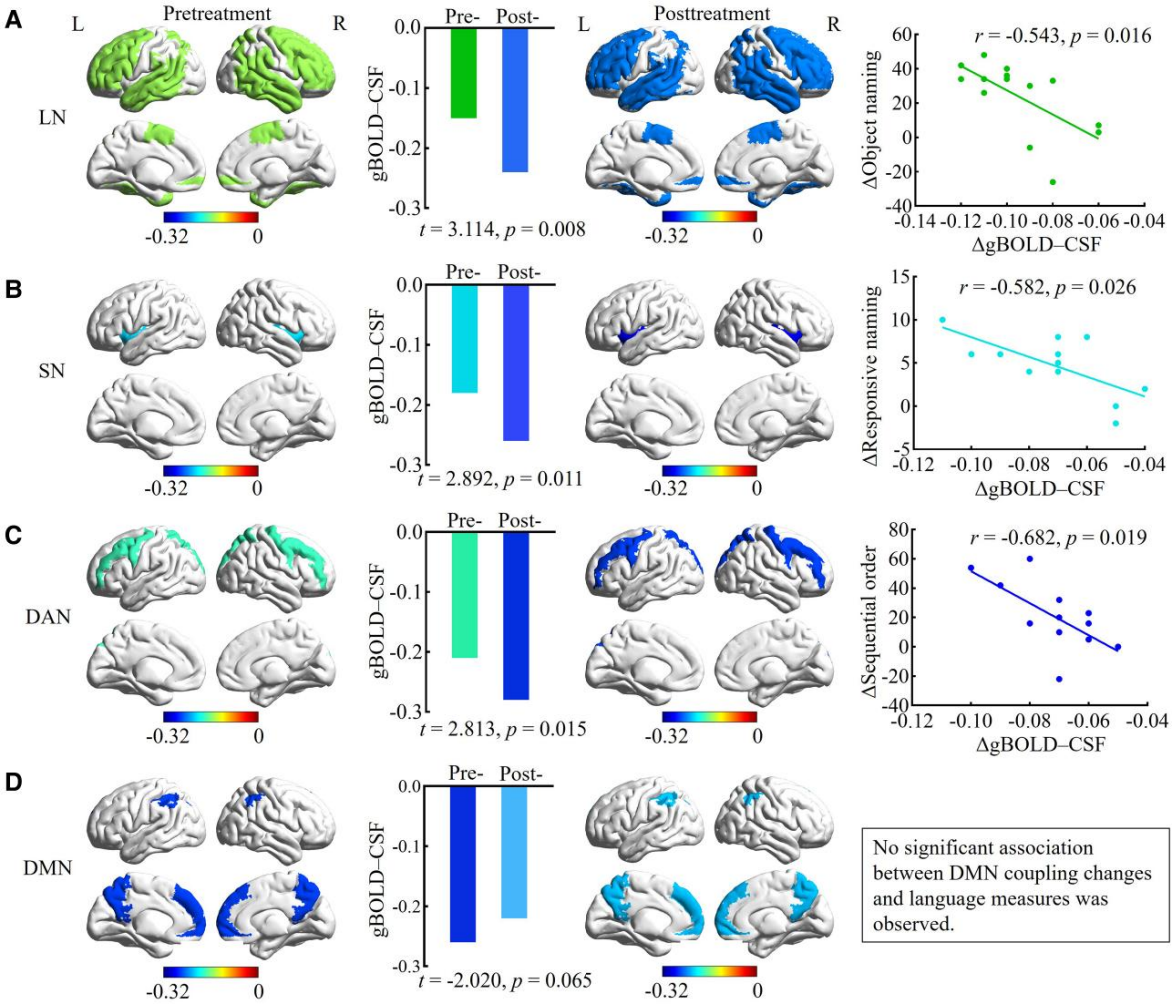
**Network-specific changes in gBOLD–CSF coupling and their associations with language recovery in patients with post-stroke aphasia.** (A–D) Surface renderings and bar graphs illustrate gBOLD–CSF coupling changes before and after treatment in four resting-state networks: LN, SN, DAN, and DMN. The left column shows the spatial extent of significant coupling before treatment. Bar graphs demonstrate significant increases in coupling following treatment (paired two-tailed *t-*test, FDR-corrected, *P* < 0.05*; n* = 14 patients). No significant association was found for the DMN (paired *t*-test, FDR-corrected, *P* = 0.065*; n* = 14 patients). The third column displays post-treatment spatial coupling changes. Rightmost scatter plots show regression analyses revealing significant negative associations between increases in coupling and improvements in object naming in LN (*r* = −0.543, *P* = 0.016), in responsive naming in SN (*r* = −0.582, *P* = 0.026), and in Sequential order in DAN (*r* = −0.682, *P* = 0.019), all FDR-corrected. Each data point represents one participant (*n* = 14). Colour bar represents gBOLD–CSF coupling values (−0.32 to 0). L = left hemisphere; R = right hemisphere; Pre = pretreatment; Post = posttreatment.

To further explore the regional specificity of treatment-related effects, we examined gBOLD–CSF coupling changes within four canonical functional networks. Coupling became significantly more negative in the SN (from −0.21 at baseline to −0.28 post-treatment, *t* = 2.892, *P* = 0.011), DAN (−0.18 to −0.26, *t* = 2.813, *P* = 0.015), and LN (−0.15 to −0.24, *t* = 3.114, *P* = 0.008), indicating stronger coupling following treatment. In contrast, coupling within the DMN became significantly less negative (−0.26 to −0.22, *t* = 2.412, *P* = 0.029) (Fig. 4), reflecting weaker coupling post-treatment. Adjusted for age, baseline performance and lesion volume, network-level regression analyses revealed significant associations between ΔgBOLD–CSF coupling and corresponding language improvements. Specifically, stronger (i.e. more negative) coupling within the LN, SN and DAN was associated with greater gains in object naming, responsive naming and Sequential order (all FDR-corrected *q* < 0.05, Fig. 4). Because gBOLD–CSF coupling values are expressed as negative correlations, greater language improvement was associated with more negative coupling values, indicating stronger synchronization between gBOLD and CSF signals. These models explained approximately 35–45% of the variance in language improvement, confirming that the observed network-specific associations were robust and independent of lesion size (Fig. 4). In contrast, whole brain coupling showed no significant correlation with overall WAB scores. Detailed baseline correlations between AQ, individual WAB subtests, and network-level gBOLD–CSF coupling indices are provided in Supplementary Table 2.

## Discussion

In this longitudinal study, we demonstrated that gBOLD–CSF coupling was significantly reduced in PSA patients compared to HCs, and that this impairment partially recovered following SLT. Notably, coupling changes showed a distinct network pattern rather than a global shift, with enhanced coupling evident in the LN, SN, and DAN. Furthermore, these network-specific increases were closely associated with improvements in corresponding language subdomains, suggesting that gBOLD–CSF coupling is responsive to both global and network-level functional plasticity during language rehabilitation.

While the glymphatic system has been extensively studied in the context of neurodegenerative diseases, its role in PSA remains poorly understood. gBOLD–CSF coupling has been proposed as a functional MRI marker of glymphatic activity, reflecting coordinated interactions between global neural activity and CSF dynamics.^16,25^ Despite growing interest in this measure, few studies have evaluated its relevance in stroke, where neurovascular regulation is often disrupted. It is therefore plausible that PSA involves impaired gBOLD–CSF coupling, but the extent of such disruption and the possibility of its reversibility through targeted rehabilitation have not been systematically examined. Our findings help fill this gap by demonstrating that coupling deficits in PSA can improve after therapy and are linked to behavioural recovery.

The reduction in gBOLD–CSF coupling among PSA patients aligns with previous observations in Parkinson’s disease and Alzheimer’s disease, where impaired CSF dynamics have been linked to cognitive and motor dysfunction.^31,32^ Following stroke, damage to neurovascular regulatory pathways may disrupt CSF pulsations, disrupt perivascular exchange and hinder metabolic clearance, thereby exacerbating neuroinflammation and delay recovery.^33,34^ Consistent with this framework, our results suggest that disrupted neurovascular–CSF communication contributes to altered brain homeostasis in stroke^35,36^ and may represent an underrecognized mechanism influencing post-stroke functional outcomes.

The post-therapy enhancement of gBOLD–CSF coupling indicates that rehabilitation may partially restore neurovascular–CSF coordination and facilitate glymphatic clearance. This is in line with prior studies showing that functional recovery after brain injury is accompanied by neuroplastic reorganization of large-scale networks, including those involved in regulating CSF flow.^37,38^ In our study, coupling increased selectively within the LN, SN and DAN—systems supporting language processing, attentional control, and cognitive flexibility.^14,39,40^ The LN is crucial for language expression and comprehension,^41,42^ and its strengthened coupling corresponded to behavioural gains, suggesting that network-specific restoration of gBOLD–CSF synchrony may underlie targeted aspects of language recovery.

The enhanced coupling within the DAN and SN is biologically plausible, as these networks mediate arousal, attention and salience detection, which are essential for goal directed behaviour after stroke.^14,39^ Greater coupling in the DAN may indicate improved neurovascular coordination supporting sequential information processing and attentional control, while increased SN coupling may reflect more efficient switching between internally and externally directed systems.^39,41^ Physiologically, stronger (i.e. more negative) gBOLD-CSF coupling in these task-positive networks may reflect restored communication between vascular and CSF systems that promotes waste clearance, metabolic efficiency and neural plasticity.^25,31,37^

In contrast, gBOLD–CSF coupling within the DMN decreased after therapy. This finding aligns with prior reports of DMN deactivation during language processing in stroke survivors^6,43^ and may reflect reduced engagement of self-referential processes during rehabilitation. Such DMN suppression likely represents an adaptive downregulation of internally focused activity, enabling greater resource allocation to task-positive systems including the LN, SN, and DAN.^43,44^ Although this rebalancing may facilitate network efficiency, we did not observe a direct correlation between DMN coupling and language performance, suggesting that DMN modulation reflects broader brain-state adjustment rather than specific functional gains.^45,46^ For instance, Gordon *et al.*^45^ showed variable DMN interaction with language and control systems, while others reported reduced DMN connectivity alongside increased LN activation,^47^ consistent with a shift towards externally oriented processing. Our results thus support the view that DMN downregulation may be adaptive, promoting recovery through dynamic reorganization of network balance.

Together, these findings underscore the network-specific plasticity of gBOLD–CSF coupling in PSA. Enhanced coordination between large-scale neural activity and CSF dynamics appears to accompany functional reorganization during therapy, linking physiological adaptation with behavioural improvement. This study provides preliminary evidence that gBOLD–CSF coupling captures dynamic neurofluid–vascular interactions underlying post-stroke recovery.

Several limitations should be noted. First, the relatively small sample size limits the generalizability and statistical power. Second, although the study was powered to detect medium-to-large effects, larger multi-centre cohorts are needed for replication. Third, glymphatic involvement should be cautiously interpreted as gBOLD–CSF coupling represents an indirect fMRI-based proxy rather than a direct measure of CSF flow and may be influenced by vascular or arousal factors. Future multi-centre investigations using advanced imaging methods such as contrast-enhanced MRI may enable more direct assessments and help clarify the role of neurofluid dynamics in language recovery.

In summary, we demonstrate that language therapy modulates gBOLD–CSF coupling in PSA, producing network-specific changes linked to distinct language improvements. These findings highlight gBOLD–CSF coupling as a physiologically meaningful and network-sensitive marker of neuroplasticity. Future multimodal imaging studies, including glymphatic-specific approaches, are needed to clarify the mechanistic contributions of fluid dynamics to post-stroke brain repair.

## Supplementary Material

fcag106_Supplementary_Data

## Data Availability

The datasets that support the findings of this study are available from the corresponding author upon reasonable request. Due to participant privacy and ethical restrictions, raw MRI data cannot be publicly shared. The custom MATLAB scripts used for data preprocessing are openly available on GitHub at: https://github.com/Freebird811/Code_for_cal_gBOLD_CSF.git.
