# Supplementary material for: Network-specific enhancement of global blood-oxygen-level-dependent signals and CSF coupling after language therapy in post-stroke aphasia
Source: Brain Commun. 2026 Mar 24;8(2):fcag106. doi: 10.1093/braincomms/fcag106 (PMC13049551; doi:10.1093/braincomms/fcag106)
Supplement: fcag106_Supplementary_Data [file fcag106_supplementary_data.docx]

**Supplementary Table 1. Correlations between lesion volume and gBOLD–CSF coupling across networks**

| Network | Baseline r (P) | Post-therapy r (P) | Δ coupling r (P) |
| --- | --- | --- | --- |
| LN | 0.12 (0.69) | –0.01 (0.98) | 0.11 (0.70) |
| SN | 0.25 (0.38) | 0.48 (0.08) | 0.44 (0.12) |
| DAN | 0.00 (0.99) | 0.15 (0.62) | 0.25 (0.39) |
| DMN | –0.16 (0.50) | –0.34 (0.24) | 0.19 (0.53) |

Note: Spearman’s rank correlations (two-tailed) were used to assess associations between lesion volume and gBOLD–CSF coupling at baseline, post-therapy, and longitudinal change (Δ = post – baseline). DAN = dorsal attention network, DMN = default mode network; gBOLD = global blood oxygen level-dependent signal; LN = language network; SN = salience network.

| **Supplementary Table 2. Partial correlations between baseline WAB scores and gBOLD–CSF coupling indices** | | | |
| --- | --- | --- | --- |
| **WAB_variable** | **Coupling_metric** | **r** | **p_FDR** |
| Object naming | ΔgBOLD–CSF_LN | 0.615 | 0.449 |
| Responsive naming | ΔgBOLD–CSF_LN | 0.612 | 0.449 |
| Naming | ΔgBOLD–CSF_LN | 0.544 | 0.471 |
| Repetition | ΔgBOLD–CSF_DAN | 0.528 | 0.471 |
| Speech Fluency | ΔgBOLD–CSF_LN | 0.491 | 0.507 |
| Spontaneous | ΔgBOLD–CSF_LN | 0.477 | 0.507 |
| Word fluency | ΔgBOLD–CSF_DAN | -0.455 | 0.507 |
| Sequential order | ΔgBOLD–CSF_DAN | 0.454 | 0.507 |
| AQ | ΔgBOLD–CSF_LN | 0.436 | 0.507 |
| Yes/no questions | ΔgBOLD–CSF_DAN | -0.431 | 0.507 |
| Speech Fluency | ΔgBOLD–CSF_SN | 0.358 | 0.685 |
| Sequential order | ΔgBOLD–CSF_SN | -0.354 | 0.685 |
| Comprehension | ΔgBOLD–CSF_SN | -0.351 | 0.685 |
| Information Content | ΔgBOLD–CSF_LN | 0.344 | 0.685 |
| Naming | ΔgBOLD–CSF_DAN | -0.295 | 0.862 |
| Comprehension | ΔgBOLD–CSF_DAN | 0.266 | 0.913 |
| Object naming | ΔgBOLD–CSF_DAN | -0.258 | 0.913 |
| Responsive naming | ΔgBOLD–CSF_DAN | -0.252 | 0.913 |
| Information Content | ΔgBOLD–CSF_DAN | -0.234 | 0.928 |
| Auditory word recognition | ΔgBOLD–CSF_SN | -0.203 | 0.928 |
| Word fluency | ΔgBOLD–CSF_SN | -0.198 | 0.928 |
| Information Content | ΔgBOLD–CSF_SN | -0.188 | 0.928 |
| Sequential order | ΔgBOLD–CSF_LN | 0.163 | 0.928 |
| Responsive naming | ΔgBOLD–CSF_SN | 0.156 | 0.928 |
| Word fluency | ΔgBOLD–CSF_LN | -0.152 | 0.928 |
| Repetition | ΔgBOLD–CSF_LN | 0.139 | 0.928 |
| Repetition | ΔgBOLD–CSF_SN | -0.137 | 0.928 |
| Yes/no questions | ΔgBOLD–CSF_SN | -0.127 | 0.928 |
| Comprehension | ΔgBOLD–CSF_LN | 0.115 | 0.928 |
| Spontaneous | ΔgBOLD–CSF_DAN | -0.114 | 0.928 |
| Sentence completion | ΔgBOLD–CSF_SN | -0.113 | 0.928 |
| Spontaneous | ΔgBOLD–CSF_SN | 0.101 | 0.940 |
| AQ | ΔgBOLD–CSF_SN | -0.052 | 0.959 |
| AQ | ΔgBOLD–CSF_DAN | 0.052 | 0.959 |
| Yes/no questions | ΔgBOLD–CSF_LN | -0.045 | 0.959 |
| Naming | ΔgBOLD–CSF_SN | -0.039 | 0.959 |
| Auditory word recognition | ΔgBOLD–CSF_DAN | 0.036 | 0.959 |
| Speech Fluency | ΔgBOLD–CSF_DAN | 0.030 | 0.959 |
| Sentence completion | ΔgBOLD–CSF_LN | -0.030 | 0.959 |
| Object naming | ΔgBOLD–CSF_SN | -0.024 | 0.959 |
| Sentence completion | ΔgBOLD–CSF_DAN | 0.023 | 0.959 |
| Auditory word recognition | ΔgBOLD–CSF_LN | 0.010 | 0.973 |
| **Note:** Pearson correlation analysis with FDR correction. AQ = Aphasia Quotient; gBOLD = global blood oxygen level-dependent signal; DAN = dorsal attention network; DMN = default mode network; LN = language network; SN = salience network. | | | |
